# Drug-Loaded Extracellular Vesicle-Based Drug Delivery: Advances, Loading Strategies, Therapeutic Applications, and Clinical Challenges

**DOI:** 10.3390/pharmaceutics18010045

**Published:** 2025-12-29

**Authors:** Linh Le Dieu, Adrienn Kazsoki, Romána Zelkó

**Affiliations:** University Pharmacy Department of Pharmacy Administration, Semmelweis University, Hőgyes Endre Street 7-9, 1092 Budapest, Hungary; linh.le@stud.semmelweis.hu

**Keywords:** extracellular vesicles, exosomes, drug delivery system, loading strategies, nanomedicine, therapeutic application, clinical translation

## Abstract

**Background/Objectives**: Extracellular vesicles (EVs) are nanosized carriers with high biocompatibility, low immunogenicity, and the ability to cross biological barriers, making them attractive for drug delivery. Despite growing interest, the clinical translation of drug-loaded EVs remains limited. This systematic review aimed to summarize current evidence on EV sources, loading strategies, therapeutic applications, and translational challenges. **Methods**: Following PRISMA 2020 guidelines, a systematic search was conducted in Embase, PubMed, Reaxys, and Scopus for the period 2020–2025. Eligible studies included original articles on drug-loaded EVs from human, animal, plant, or other sources. Data on EV source, drug type, particle size, loading method, administration route, and therapeutic application were extracted. Clinical trials were identified through ClinicalTrials.gov. **Results**: A total of 65 studies were included after screening 5316 records, along with two clinical trials. Human mesenchymal stem cell (MSC)-derived EVs were the most frequent source in oncology, while plant-derived EVs predominated in non-oncology applications. Anti-cancer drugs such as doxorubicin, gemcitabine, and docetaxel were most frequently loaded, alongside curcumin, berberine, and atorvastatin. EV sizes generally ranged from 50 to 200 nm, with larger vesicles reported for plant-derived EVs. Intravenous administration predominated, with most studies demonstrating sustained release and enhanced therapeutic efficacy. Passive loading was most common, especially for hydrophobic drugs, whereas active methods such as electroporation and sonication were preferred for hydrophilic cargo. Two clinical trials showed preliminary therapeutic benefits with favorable safety. **Conclusions**: Drug-loaded EVs represent a promising and versatile drug delivery platform, yet their clinical translation is hindered by variability in isolation and loading methods, production scalability, and safety evaluation. Further standardization and large-scale studies are needed to advance EV-based therapeutics toward clinical use.

## 1. Introduction

Extracellular vesicles (EVs) are nanosized lipid bilayer vesicles secreted by nearly all cell types, mediating intercellular communication through the transfer of proteins, nucleic acids, lipids, and metabolites [1,2]. EVs are broadly divided into two main subtypes based on their biogenesis: exosomes, which are generated within multivesicular bodies and released upon their fusion with the plasma membrane (30–150 nm), and microvesicles (ectosomes), which are formed by direct outward budding of the plasma membrane (100–1000 nm). Their particle size typically ranges from 50 to 200 nm, although larger vesicles up to 350–400 nm have also been reported, particularly in plant-derived EVs [3,4]. Apoptotic bodies represent another class of EVs, but they are less commonly investigated for drug delivery applications. Because of their natural origin, EVs exhibit several desirable features for biomedical applications, including high biocompatibility, low immunogenicity, and the ability to cross biological barriers such as the blood–brain barrier [5]. These properties have spurred increasing interest in harnessing EVs as drug delivery systems.

Drug-loaded EVs offer several advantages over conventional synthetic nanocarriers (e.g., liposomes, polymeric nanoparticles, micelles). They can protect therapeutic cargo from enzymatic degradation, prolong circulation time, and facilitate targeted delivery through surface molecules inherited from their parent cells [6,7]. Moreover, EVs can encapsulate a diverse range of therapeutic agents, including small molecules such as doxorubicin, curcumin, and cisplatin, as well as proteins and nucleic acids; however, in the 2020–2025 studies meeting our criteria, exogenous loading was applied predominantly to small-molecule cargo [2,8,9]. Preclinical studies have demonstrated promising applications in oncology [2,9,10], neurodegenerative diseases (Alzheimer’s) [11], metabolic disorders (diabetes) [12,13], infectious diseases [4,14], rheumatoid arthritis [15,16], and tissue regeneration [17], while early-phase clinical trials are beginning to test their feasibility in humans (NCT04879810 [18]; NCT06930326 [19]).

Despite this potential, several barriers continue to hinder the clinical translation of drug-loaded EVs. There is no consensus on standardized protocols for EV isolation, characterization, and drug loading, leading to heterogeneity across studies [2,20,21,22]. Drug loading strategies vary considerably, from simple passive incubation [8] to active methods such as sonication and electroporation [2,9,20] each with trade-offs in efficiency and vesicle integrity. Large-scale production and quality control remain challenging due to the biological complexity of EVs [7,23], and safety concerns such as long-term biodistribution and immunogenicity are insufficiently addressed [24,25,26].

While previous reviews have discussed the biological properties of extracellular vesicles more broadly [16,27,28], this systematic review provides a focused, evidence-based synthesis emphasizing: (1) comprehensive EV source comparison, including human, animal, plant, and bacterial sources [29]; (2) a mechanism-of-action framework with a 3-step cellular uptake model; (3) disease-specific pathophysiological targeting strategies for oncology, neurodegeneration, and inflammatory disorders; (4) critical analysis of active versus passive loading controversies; (5) manufacturing scalability solutions with cost–benefit analysis [30]; and (6) regulatory pathway guidance for bioengineered EV platforms [31].

Therefore, this review aims to provide a comprehensive overview of drug-loaded EVs published between 2020 and 2025, examining EV sources, drug-loading strategies, particle characteristics, administration routes, therapeutic applications, and the status of clinical translation. By consolidating current evidence, we seek to highlight both the opportunities and the challenges in advancing EV-based drug delivery toward clinical application.

In contrast to existing reviews, the present work integrates EV source comparison, loading strategy trade-offs, and a matrix-based evaluation of loading efficiency to provide a translationally focused framework for drug-loaded EV-based delivery systems.

## 2. Materials and Methods

The Preferred Reporting Items for Systematic Reviews and Meta-Analyses (PRISMA 2020 in Supplementary Materials) guidelines were used to conduct the search for relevant studies and reports. In addition, interventional clinical trials were identified through ClinicalTrials.gov using the same search strategy and eligibility criteria.

### 2.1. Eligibility Criteria

Studies involving extracellular vesicles (EVs) derived from humans, animals, plants, or other sources loaded with therapeutic agents were eligible for inclusion. Eligible drug categories included: (1) small-molecule drugs (doxorubicin, curcumin, cisplatin); (2) biologics (antibodies, proteins, insulin); (3) nucleic acid therapeutics (siRNA, miRNA, plasmid DNA, mRNA) [32,33]; and (4) phytochemicals (berberine, curcumin, astragalus components). Although nucleic acid therapeutics (siRNA, miRNA, plasmid DNA, mRNA) were included in the eligibility criteria, the final dataset was numerically dominated by EVs loaded with small-molecule drugs, with only a limited number of studies reporting exogenous loading of nucleic acid payloads. Only original research articles published in peer-reviewed journals were included. Reviews, editorials, conference abstracts, protocols, and commentaries were excluded.

Clinical trials were included if they investigated extracellular vesicles derived from human, plant, or other biological sources that were actively loaded with a therapeutic agent and administered to human participants. Both interventional and completed trials were eligible, provided that drug loading into EVs was explicitly described. Trials using EVs as biomarkers or without exogenous drug loading were excluded. Ongoing, terminated, or withdrawn studies without available results were not included in the analysis.

### 2.2. Search Strategy

A comprehensive systematic search was conducted across Embase, PubMed, Reaxys, and Scopus. The search strategy employed a combination of keywords as follows: Extracellular vesicles AND drug delivery OR drug-loaded extracellular vesicles. The search interval was between January 2020 and September 2025. Only articles written in English were included.

Clinical trials were searched exclusively in ClinicalTrials.gov using the same combination of keywords. Trials were included only if they investigated exogenously drug-loaded EVs, were registered as interventional studies, and reported at least summary results; trials using unmodified EVs, EV-based diagnostics, or without a clearly described loading step were excluded. Under these strict criteria, two completed clinical trials were identified and analyzed.

### 2.3. Screening Process and Data Extraction

All the records identified from different databases were imported into EndNote for screening, and then duplicate records were removed. The articles were initially screened based on their titles and abstracts, followed by a full-text review of all those deemed eligible.

The extracted data was organized into three structured tables (Table 1, Table 2 and Table 3).

- Table 1 summarizes the characteristics of each study, including the type and source of EVs, type of loaded drug, particle size, targeted release of the active pharmaceutical ingredient (API), route of administration or drug release characteristics, and therapeutic indication.
- Table 2 presents detailed information on the type of loaded drug, loading method, loading efficiency, loading conditions (e.g., pH value, added excipients), and observed outcomes.
- Table 3 provides data on the clinical trials, including the type of vesicle, trial status, indication, loaded active ingredient, observed outcomes, and the ClinicalTrials.gov identifier (NCT number).

## 3. Results

### 3.1. Study Selection

**Figure 1:**
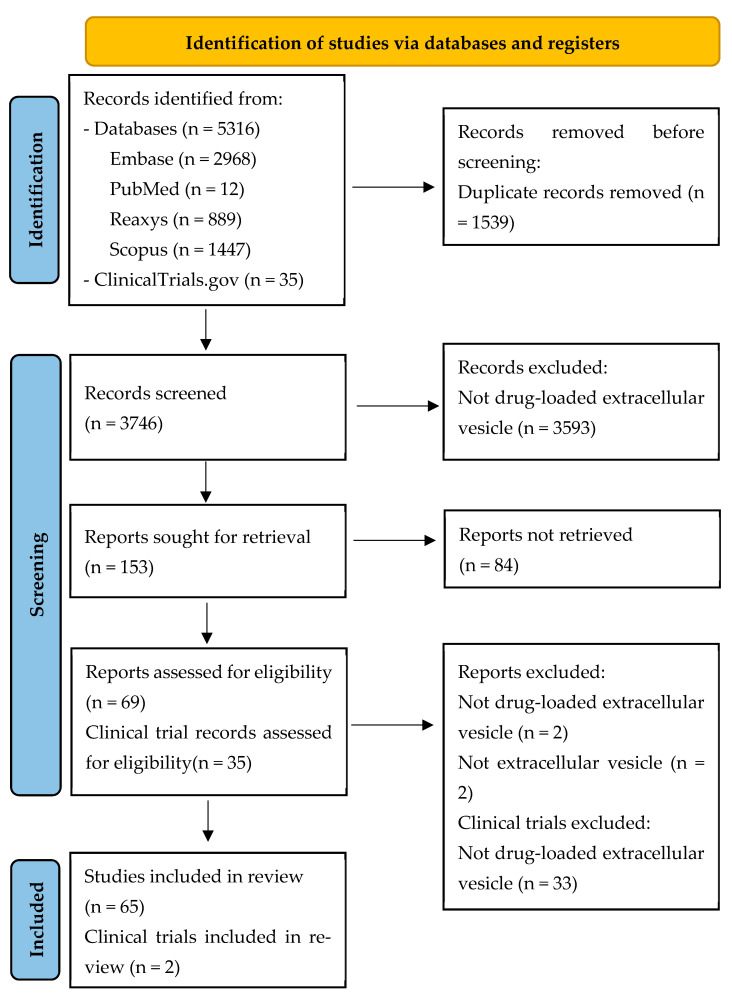
PRISMA 2020 flow diagram of the study selection process for drug-loaded extracellular vesicle studies.

A total of 5316 articles were identified from different databases, including Embase, PubMed, Reaxys, and Scopus. 2968 were from Embase, 12 from PubMed, 889 from Reaxys and 1447 from Scopus. After screening, at the end, 64 articles were included in the review. 2 clinical trials that met the inclusion criteria from clinicaltrials.gov were also selected for this review. The detailed selection process is illustrated in Figure 1, following the PRISMA 2020 flow diagram.

### 3.2. Overview of Included Studies

The data extracted from the included studies were compiled into four main tables summarizing the characteristics and outcomes of drug-loaded EVs. Table 1 provides an overview of the EV sources, types of loaded drugs, particle size, administration route, and therapeutic indications. Table 2 presents the main loading strategies, efficiencies, and experimental conditions, while Table 3 summarizes completed clinical trials involving drug-loaded EVs. Within the narrow scope of exogenously drug-loaded EVs registered in ClinicalTrials.gov, two completed clinical trials with available outcomes were identified (Table 3). This focused subset does not encompass the broader clinical EV landscape, which includes numerous trials of unmodified or endogenously loaded EV therapeutics summarized in recent reviews. The following subsections describe the main findings derived from these data. Table 4 presents the Biopharmaceutics Classification System analysis with loading efficiency data. The following subsections describe the main findings derived from these data.

#### Comparative Analysis of EV Sources

Extracellular vesicles used in drug delivery research derive from diverse biological sources, each with specific advantages, limitations, and regulatory implications [34]. A comparative analysis of human-, animal-, plant-, milk-, and bacterial-derived EVs reveals distinct profiles with respect to yield, scalability, immunogenicity, and translational potential.

Human-derived EVs, particularly from mesenchymal stem cells (MSCs), dominated preclinical oncology studies. These vesicles inherit cell-type-specific surface markers (e.g., CD29, CD44, CD90) that can confer organotropism and tumor-homing capacity. However, production yields from conventional 2D MSC cultures are modest (typically 0.1–0.5 × 10^9^ EVs/mL of conditioned medium), and batch-to-batch variability can be substantial (15–35% CV). Despite these limitations, human-derived EVs benefit from the most mature regulatory experience.

Animal-derived EVs (e.g., bovine, equine, porcine sources) offer moderate scalability (1–2 × 10^9^ EVs/mL) and lower costs compared with human primary cells. However, zoonotic safety concerns and less clearly defined regulatory pathways may complicate clinical development.

Plant-derived EVs from edible plants (e.g., ginger, grapes, grapefruit) represent a rapidly growing category. These vesicles can be produced at large scale from agricultural by-products, exhibit excellent stability (6–12 months at refrigerated conditions), and typically demonstrate low immunogenicity. The main limitations are compositional heterogeneity and uncertain regulatory classification.

Milk-derived EVs leverage a strong dietary safety precedent. They occur at very high concentrations (10^9^–10^10^ EVs/mL), are scalable via dairy-industry infrastructure, and are promising for oral or mucosal delivery. However, regulatory classification as food vs. therapeutic biologic is not yet fully harmonized.

Bacterial-derived EVs (e.g., outer membrane vesicles from Gram-negative bacteria) emerge as immunomodulatory platforms. They offer high yields via fermentation (5–10 × 10^9^ EVs/mL) and structural robustness, but concerns about endotoxin content and pyrogenicity require stringent purification strategies.

From a cost–benefit perspective, human EVs incur the highest production costs per dose (tens of thousands to >100,000 USD), whereas plant- and milk-derived EVs have the lowest costs due to abundant raw materials. Timelines to regulatory approval are shortest for human- and milk-derived vesicles, intermediate for animal and plant EVs, and longest for bacterial-derived EVs.

**Table 1:** Summary of drug-loaded EV studies (2020–2025).

Type of EVs Source	Source of EVs	Type of Loaded Drug	Particle Size (nm)	Targeted Release of API	Administration Route/Drug Release Characteristic	Indication	Reference
Human	Periodontal ligament stem cells (PDLSCs)	Simvastatin	30–150	Alveolar bone	- -	Relapses after orthodontic tooth movement (Bone regeneration)	[35]
	Activated T cell	Paclitaxel-poly-L-lysine prodrug	123 ± 7	Tumor cell	- i.v - Sustained	Triple-negative breast cancer	[23]
	Mesenchymal stem cells	Rifampicin	65–225	Tumor cell	- i.v - Sustained	Osteosarcoma	[1]
	T cell	Doxorubicin	146–147	Tumor cell	- i.v - Sustained	Tumor treatment	[36]
	Non-small cell lung carcinoma A549	Doxorubicin and lonidamine	50–200	Cancer cell	- i.v - Sustained	Lung cancer	[2]
	Endometrial mesenchymal stem cells	Atorvastatin	50–200	Glioblastoma tumor cell	- -	Glioblastoma	[37]
	Mesenchymal stem cells (MSCs)	Doxorubicin	169	Tumor cell	- Intraperitoneal - Sustained	Colorectal cancer	[6]
	Periodontal stem cells	Aspirin	206	Macrophage	- Intraperitoneal -	Periodontitis	[38]
	Mesenchymal stem cells (MSCs)	Baricitinib	120	Hair follicle	- i.v -	Alopecia areata	[24]
	M2-type macrophage	Berberine	125 ± 12	Injured spinal cord	- Intraperitoneal - Sustained	Spinal cord injury	[7]
	Mesenchymal stem cells (MSCs)	Berberine	103	Injured spinal cord	- - Sustained	Spinal cord injury	[39]
	HepG2 tumor cell	Bleomycin	105	Tumor cell	- i.v -	Cancer	[25]
	Primary M2 macrophage	Curcumin	124	Site of inflammation	- i.v -	Spinal cord injury and rheumatoid arthritis	[8]
	Mesenchymal stem cells (MSCs)	Cisplatin	139 ± 47	Tumor cell	- -	Cervical cancer therapy	[9]
	Fibroblast	Clodronate	120	Fibroblast	- i.v -	Pulmonary fibrosis	[40]
	A549 cancer cells	Docetaxel	94–250	Tumor cell	- i.v -	Lung cancer	[41]
	M1 macrophage	Docetaxel	163	Tumor cell	- -	Breast cancer	[42]
	Plasma	Donepezil	107	Brain	- i.v - Sustained	Alzheimer	[11]
	Breast cancer cell lines (4T1 and SKBR3)	Doxorubicin	149–200	Tumor cell	-	Retinoblastoma	[43]
	Endothelial cell	Doxorubicin	50–200	Glioma cell	- i.v -	Glioblastoma	[44]
	Natural killer cell	Doxorubicin	131	Tumor cell	- -	Triple-negative breast cancer	[45]
	SKOV3 ovarian cancer cells	Curcumin	100	Tumor cell	- -	Ovarian cancer	[21]
	JAWS II dendritic cell	Delphinidine	117 ± 2	Aortic endothelial cell	- -	Antiangiogenic	[22]
	Immature dendritic cells	Berberine chloride	1106 ± 12	Tumor cell	- -	Antitumor	[46]
	RAW 264.7 macrophages	Lyostaphin and vancomycin	96 ± 6	Liver and spleen	- i.v - Sustained	Intracellular MRSA	[47]
	Mesenchymal stem cells	Rapamycin	<200	Brain	- i.v - Sustained	Glioblastoma	[26]
	RAW 264.7 cells	Docetaxel	122 ± 1	Tumor cell	- - Sustained	Breast cancer	[48]
	Bone marrow mesenchymal stem cells	Doxorubicin	30–200	Bone tumor	- i.v - Sustained	Osteosarcoma	[49]
	Hepatocellular carcinoma	Insulin	153 ± 64	Pancreatic islet	Sustained	Diabete	[12]
	Adipose-Derived Stem Cells	Curcumin	50–200	Skin flap	- i.v -	Flap grafting survival	[17]
	Pluripotent stem cells	Amphotericin B		Brain	- i.v -	Cryptococcal meningitis	[50]
	Keratinocyte	Tofacitinib	71 ± 3	Keratinocytes	- -	Psoriasis	[51]
	Placental mesenchymal stem cells	Doxorubicin	30–200	Breast cancer cell	- i.v -	Breast cancer	[52]
	Bone marrow mesenchymal stem cells	Luteolin	200	Liver cell	- Intraperitoneal - Sustained	Liver fibrosis	[53]
	Mesenchymal stem cells	Doxorubicin	142–178	Bone	- i.v -	Osteosarcoma	[54]
	Platelet	Kaempferol	144	Eye	- - Sustained	Corneal neovascularization	[55]
	Mesenchymal stem cells	Bevacizumab	155 ± 6	Eye	-	Diabetic retinopathy	[56]
	Mesenchymal stem cells	Rapamycin	50–200	Eye	-	Autoimmune Uveitis	[5]
	Embryonic kidney HEK293 cell	Melatonin	100	Skin	-	Atopic dermatitis	[57]
	Metastatic murine melanoma cells	Zinc-phthalocyanine	100–200	Tumor cell	- i.v/intratumoral -	Colon cancer	[58]
	Bone marrow mesenchymal stem cells	Gemcitabine	60–100	Pancreatic cell	- -	Pancreatic cancer	[59]
	Umbilical cord blood-mesenchymal stem cell	Docetaxel	218 ± 17	Tumor cell	- - Sustained	Cancer	[60]
	Breast cancer cell MDA-MB-231	Doxorubicin	140 ± 3	Breast cancer cell	- i.v -	Breast cancer	[61]
	HEK293 cells	Curcumin	334 ± 3	-	- -Sustained	-	[62]
	SF7761 stem cells-like- and U251-GMs	Doxorubicin	100	Glioma cell	- -	Malignant glioma	[63]
Animal	Mouse BMSCs	Rifampicin	157 ± 4	Brain	- i.v -	Central nervous system tuberculosis	[64]
	Milk	Anthocyanin	106	HepG2 cell	-	Antitumor	[65]
	Milk	Dexamethasone	70	Inflammatory cell	- - Sustained	Corneal alkali burn	[66]
	Mouse platelet	Resveratrol	114 ± 8	Injured site	- - Sustained	Promoting wound healing in diabete mice	[67]
	Animal milk and human mesenchymal stem cells	Doxorubicin	114 ± 3	Tumor cell	- -	Cancer	[68]
	Mouse glioma C6 cells	Cetuximab and doxorubicin	125 ± 6	Brain	- i.v - Sustained	Glioblastoma	[69]
	Mouse macrophage	Atovaquone	84	Tissue cyst	- i.v -	Toxoplama gondii infection	[14]
	Milk	Glycyrrhetinic acid	122 ± 2	Lung	- i.v - Sustained	Idiopathic pulmonary fibrosis	[70]
	Milk	Oxaliplatin	50–200	Tumor cell	- i.v - Sustained	Cancer	[71]
Plant	Banana	Curcumin	50–250	Colon	- -	Inflammatory bowel disease	[72]
	Curcumin	Doxorubicin	120–190	Tumor cell	- i.v - Sustained	Cancer	[73]
	Grapefruit	Sodium thiosulfate	196 ± 18	Site of vascular calcification	- Intraperitoneal - Sustained	Vascular calcification	[74]
	Curcumin	Astragalus component	154	Tumor cell	- - Sustained	Antitumor	[75]
	Ginger	Curcumin	<400	Colon	- Oral (gavage) -	Anti-colitis	[3]
	Plant Kaempferia parviflora	Clarithromycin	352 ± 23	GI tract	- - Sustained	*Helicobacter pylori* Infection	[4]
Other	Probiotic *Lactobacillus* species	Doxorubicin	142 ± 1	Bacteria	- - Sustained	Staphylococcus species	[76]
	Artificial cell NK cell line NK-92	Docetaxel	152 ± 17	Tumor cell	- -	Lung cancer	[77]
	Plasma	Quercetin	150	Brain	- i.v -	Alzheimer	[78]
	Transferrin-modified SPIONs	Quercetin	85	Pancreatic islet	- i.v -	Type 2 diabetes	[13]

**Table 2:** Summary of drug-loaded EV studies (2020–2025).

Type of Loaded Drug	Type of Loading	Loading Efficacy	Loading Circumstances (pH Value, Added Excipient…)	Outcome	Reference
17ß-Estradiol	Passive + active	Passive: 75 ± 4 ng/100 µg Active: 60 ± 3 ng/100 µg	- Incubation: 37 °C for 1 h - Sonication: 20% amplitude and 6 cycles of 30 s on/off for 3 min with a 2 min cooling period between each cycle	Significantly increased survival rate of BMMSCs (Bone marrow mesenchymal stem cells) treated with 17β-estradiol-loaded exosomes in comparison with the control group	[20]
Paclitaxel-poly-L-lysine prodrug	Active	-	Membrane extrusion: 220 nm polycarbonate membrane	Mediates the synergistic effects of gene therapy and chemoimmunotherapy	[23]
Rifampicin	Active + passive	-	- Sonication: 20% amplitude, 6 cycles of 30 s on/off for three minutes with a two-minute cooling period between each cycle - Incubation at 37 °C for 60 min	Display potent antitumor therapeutic effects with remarkably low toxicity	[1]
Doxorubicin	Passive	-	Incubation in 37 °C and 50 °C water baths for 2 h	Efficient drug delivery capability in vitro and in vivo	[36]
Doxorubicin and lonidamine	Passive	1 ± 0.2% and 4 ± 1.9% (Encapsulation efficiency)	Incubation overnight at 4 °C	Enhancement in anti-cancer efficacy both in vitro and in vivo	[2]
Atorvastatin	Active	-	Incubation along with Tween-20	Enhanced antitumor effects compared to free atorvastatin	[37]
Doxorubicin (DOX)	Active	199 ng 13% Encapsulation efficiency	Excipient: trimethylamine	The functionalization of the surface of exosomes with AS1411-DNA aptamer molecules considerably expanded the binding affinity and uptake rate in nucleolin-positive cells	[6]
Aspirin	Active	0.6 μg mL^−1^	Sonication: intermittent for 2 min on ice	Able to restore a certain degree of alveolar bone loss caused by periodontitis	[38]
Baricitinib	Active	86 μg mL^−1^	Electroporation: 125 μF, 250 V, Max capacitance, 10 pulses with a 2 s interval	Improvement of drug delivery efficiency, as well as the synergistic effect of EVs	[24]
Berberine	Passive + active	-	- Passive: incubation at 37 °C for 2 h - Active: sonication for 30 s in an ice bath	Accomplish the accumulation of Ber at high concentrations in the injury site	[7]
Berberine	Passive + active	-	- Passive: incubation at 37 °C - Active: sonication for 30 s with a 30 s pause for a small cycle, and after 3 small cycles with a 3 min pause	Decrease the level of local inflammation and the fiber extent of spinal cord injury in rats	[39]
Bleomycin	Passive	-	Incubation 20 min at 37 °C	Enhanced delivery of the drug to tumor cells	[25]
Curcumin	Active	28% Encapsulation efficiency	Sonication	Improve motor function in inflammation models	[8]
Cisplatin	Passive	32 ± 3% Loading efficiency	Incubation: 1 h at 37 °C in a water-bath shaker	Improved therapeutic efficacy in hampering cervical cancer progression both in vitro and in vivo	[9]
Clodronate	Active	71 ± 3% Encapsulation efficiency	- Reverse phase evaporation method - Sonication: 30% amplitude, 30 s pulse on/off, for 2 min	Significantly enhanced pulmonary fibrotic drug delivery	[40]
Docetaxel	Active	12 ± 6% Encapsulation efficiency	Electroporation: five mild electric shocks at 0.75 V in 15 s intervals	Increased drug potency compared to that of the free DTX.	[41]
Docetaxel	Active	18 ± 3% Drug loading efficiency	Electroporation: 4 mm path length cuvettes	Improved the anti-cancer therapeutic efficacy with minimal side effects	[42]
Donepezil	Active + passive	43 ± 0.8 Drug loading efficiency	- Sonication in Milli-Q water (250 μL) for 5 min - Incubation at 37 °C for 60 min	Higher pharmacological response, lower peripheral side effects, and without toxicity	[11]
Doxorubicin	Passive	82 × 10^−11^ µM Dox/EV	Incubation	Display improved cellular internalization over free Dox	[43]
Doxorubicin	Passive	-	Oil-in-water emulsion, followed by solvent evaporation and dialysis	Facilitate penetration of anti-cancer drugs across the BBB (blood–brain barrier) and target the GBM (glioblastoma)	[44]
Doxorubicin	Active + passive	51 ± 0.4% Drug loading efficiency	- Ultrasonication: 20% amplitude, six cycles of 30 sec on/off with 2 min cooling period between each cycle - Incubation at 22 °C for 120 min	Readily be engineered to target specific markers	[45]
Curcumin	Passive + active	Drug loading efficiency: - 10% for the incubation method - 11% for the sonication method - 9% for the freeze–thaw cycling method.	- Incubation: 1 h at 37 °C in the dark - Sonication: 6 cycles of 30 s - Freeze–thaw cycling: alternatively frozen at −70 °C and then thawed at room temperature	High stability and safety for delivery of drugs such as curcumin, but low loading efficiency of drug	[21]
Delphinidin	Passive	9% Drug loading efficiency	- Mix and stir	Protect delphinidin and its metabolites from degradation	[22]
Berberine hydrochloride	Active + passive	42 ± 2% Encapsulation efficiency	- Sonication for 5 min - Incubation at 37 °C for 120 min	Increase the efficacy of free BRB	[46]
Lyostaphin and vancomycin	Active + passive	-	- Sonication: 20% power, 10 cycles of 4 s pulse/2 s pause - Incubation at 37 °C for 60 min	Employ the mannosylated exosomes to deliver lysostaphin and vancomycin to bacterial infection sites to eradicate intracellular MRSA	[47]
Rapamycin	Passive	52% Encapsulation efficiency	Incubation: 37 °C for 2.5 h	Exhibit faster and more efficient release at tumor sites	[26]
Docetaxel	Passive	78% Encapsulation efficiency	Vortex	Enhancing the cellular uptake	[48]
Doxorubicin	Active	-	Excipient: Ammonium sulfate	Excellent antitumor properties both in vivo and in vitro	[49]
Insulin	Active	50 ± 4% Drug loading efficiency	Electroporation at 200 V and 50 µF in 0.2 cm Invitrogen electroporation cuvettes	Insulin-loaded exosomes were internalized by their respective donor cells and were able to promote and enhance the transport and metabolism of glucose	[12]
Curcumin	Active	-	Excipient: Methanol	Enhance its duration and effect in the localization of skin flaps	[17]
Amphotericin B	Active + passive	-	- Co-incubation: at 37 °C for 2 h in the dark - Ultrasound: 37 kHz, 30 % power, pulse sonication for 15 min - Extrusion: 10 nm pore size - Electroporation: use the Loenza Amaxa 4D Nucleofactor	At least an eightfold increase in antifungal efficacy in vitro	[50]
Tofacitinib	Active + passive	-	- TFC incubation with donor cells of exosomes - TFC incubation with exosomes - Freeze–thaw cycles: frozen at −80 °C then brought back to room temperature, repeat 3 times - Probe sonication: 500 v, 2 kHz, 20% power, 6 cycles by 4 s pulse/2 s rest - Ultrasonic bath: for 20 min	Help us in diseases where communication between cells is the determining factor in the disease	[51]
Doxorubicin	Active	-	Ultrasonic: 20% amplitude with a 30 s on/off cycle	Significantly reduced cardiac toxicity and prolonged effectiveness	[52]
Luteolin	Passive + active	- Incubation: 3 ± 0.1% - Sonication: 40 ± 1% Encapsulation efficiency	- Passive: incubation at 37 °C - Active: sonication at 20% amplitude, 10 cycles of 3 s on/off for 3 min with 2 min cooling period in an ice bath between each cycle	Augmenting drug effect	[53]
Doxorubicin	Active	-	Excipient: triethylamine	Enhance toxicity against osteosarcoma and less toxicity in heart tissue	[54]
Kaempferol	Passive	61 ± 5% Encapsulation efficiency	Mixing	Show a synergistic effect	[55]
Bevacizumab	Active + passive	- Freeze–thaw cycle: 61 ± 6 µg - Co-incubation at RT: 65 ± 3 µg - Saponin treatment: 74 ± 6 µg - Sonication: 61 ± 6 µg	- Freeze–thaw cycle: incubated at RT for 30 min, followed by freezing at −80 °C for 30 min; repeat 3 times - Incubation at room temperature - Incubation with 0.2% saponin - Sonication: 500 v, 2 kHz, 20% power, 4 cycles, 4 s pulse, and 2 s pause	Reduce the frequency of intravitreal injection required for treating diabetic retinopathy	[56]
Rapamycin	Active	-	Sonication: 25% power, 6 cycles of a 30 s pulse/30 s pause	Low risk of immunogenicity and tumorigenicity compared to cells	[5]
Melatonin	Passive	97 ng/µg	Extrusion: three-step extrusion process through 10-, 5-, and 1-μm polycarbonate membrane filters	Ease of displaying targeting molecules on the surface of NVs when the cells are genetically engineered to express the targeting molecules	[57]
Zinc-phthalocyanine	Passive	-	Incubation	Slightly promoted the apoptosis of cancer cells	[58]
Gemcitabine	Active + passive	Drug loading efficiency: - 0.6 ± 0.2% for the incubation method - 4 ± 0.4% for the sonication method - 4 ± 0.5% for the electroporation method	- Electroporation: 2 mm cuvette - Ultrasonication: 20% amplitude for 30 s for three cycles with an interval of 90 s of ice cooling - Co-incubation: for 96 h	Potent cytotoxicity against pancreatic cancer cells	[59]
Docetaxel (DTX)	Passive + active	9 ± 2 DTX(ng)/exosomes (μg)	- Passive: incubation for 2 h at 37 °C - Active: sonication at amplitude of 15% for 4 cycles	Superior in cytotoxicity in comparison to free DTX, with almost twice the potency	[60]
Doxorubicin	Active	89 ± 2% Encapsulation efficiency	Excipient: Ammonium sulfate	The association of EVs in the lipid bilayer does not impair sensitivity to acidic pH	[61]
Doxorubicin	Active	-	Excipient: SF7761 GMs-derived exosome	Inhibit tumor growth in a mouse model of glioma by its delivery through the olfactory route with a nasal spray formulation	[63]
Rifampicin	Active	-	- Electroporation	Exhibit excellent brain targeting ability in vitro and vivo	[64]
Anthocyanin (ACN)	Active + passive	-	- Ultrasonic - Electroporation - Saponin - Incubation - Freeze–thaw cycles	The formulation prepared using the ultrasonic method can effectively enhance the stability of ACN	[65]
Dexamethasone	Active	-	Ultrasonication	Increased targeting affinity for macrophages	[66]
Resveratrol	Passive	-	Incubation: 37 °C for 1 h	Can be used as wound dressing for sustained drug release	[67]
Doxorubicin	Passive + active	-	- Incubation: 37 °C for 2 h - Electroporation: using a Neon™ Transfection System - Sonication: 20% amplitude, and 6 cycles of 30 s on/off for 4 min with a 2 min cooling period between each cycle	With electroporation, greatest success was reached in loading Dox into the EVs and minimal negative effects on surface proteins were detected, while sonication seemed to be detrimental to these proteins	[68]
Cetuximab	Passive	-	Incubation: 40 °C for 2 h	Synergistically deliver antibodies and drugs into the brain via intravenous administration	[69]
Atovaquone	Passive	57 ± 6% Encapsulation efficiency	Incubation: 12 h at room temperature under stirring	Elicit potent anti-toxoplasmosis activity	
Glycyrrhetinic acid	Passive	9% Drug loading efficiency	- Co-incubation: 37 °C for 1 h	Enhance lung function recovery	[70]
Oxaliplatin	Passive	-	- Incubation: 12 h at 4 °C	Have a high potential for treating solid tumor	[71]
Curcumin	Active	-	- pH-driven method: pH = 12	Increase the bioavailability of hydrophobic drugs	[72]
Doxorubicin	Passive	38 ± 2% Drug loading capacity	- Co-incubation: Curcuma and doxorubicin in 1:1 ratio at 37 °C and 200 rpm for 2 h	The nanoparticles exhibit excellent stability and controlled drug release properties	[73]
Sodium thiosulfate	Passive	-	Incubation at room temperature for 24 h	The nanodrugs exhibit excellent cellular uptake capacity	[74]
Astragalus component	Active	34% Encapsulation efficiency	Sonication: 50 Hz, 30 min, 100%	Enhancement of synergistic antitumor activity.	[75]
Curcumin	Active + passive	89± 0.3% Encapsulation efficiency	- Ultrasound: 20% amplitude for 1 min per cycle - Incubation: 37 °C for 1 h	Shows better *anti-*ulcerative colitis activity than either free curcumin or ginger-derived nanovesicles	[3]
Clarithromycin (CLA)	Passive	92 ± 4% Encapsulation efficiency	Incubation at room temperature for 1 h on a rotator	KPEVs-CLA (*Kaempferia parviflora* extracellular vesicles) showed superior anti-inflammatory activity	[4]
Doxorubicin (DOX)	Passive + active	Encapsulation efficiency: - Shaking: 58 ± 4% - Electroporation: 63 ± 6% - Sonication: 72 ± 5%	- Shaking: 200 rpm and 37 °C for 4 h - Electroporation: single pulse of 1.8 kV - Sonication: 120 W, 30 kHz, 20% amplitude, and 30 cycles of 2 s interval on/off time	DOX_LEV(lactic acid bacteria-derived Evs)_ exhibited enhanced antibacterial activity against Staphylococcus species compared to DOX_free_	[76]
Docetaxel (DTX)	Active	1% Encapsulation efficiency	Extrusion: 100 μg/mL of Docetaxel is added prior to extrusion	Increase the efficacy of DTX	[77]
Quercetin	Passive	30 ± 8% Encapsulation efficiency	Incubation: continuous shaking at 4 °C overnight	Enhance the bioavailability of Quercetin	[78]
Quercetin	Passive	-	Incubation for 8 h at 4 °C	Better stability and higher solubility	[13]
Curcumin	Passive	-	Incubation for 30 min, at 37 °C, at neutral pH with shaking	Effective EV–curcumin delivery system with good stability, release control, and cytocompatibility	[62]

**Table 3:** Summary of drug-loaded EVs clinical trials (2020–2025).

Type of Vesicle	State of Clinical	Condition	Loading Active	Outcome	NCT Number
Exosome	Completed	Male pattern baldness	Ecklonia cava (brown seaweed) Thuja orientalis (medicinal plant)	Inclusion criteria: - Malaysian men aged 20–50 - Diagnosed with Norwood Grade 2–3 androgenic alopecia Exclusion criteria: - Have thyroid issues, bleeding disorders, or diabetes - Use medical hair treatments, steroids, or immunosuppressants - Have Norwood Grades 1, 4–7, or cicatricial alopecia - Smokers Result: hair density and thickness improvements with minimal reported side effects	NCT06930326 [19]
Plant Exosomes	Completed	Inflammatory Bowel Disease	Curcumin	Inclusion criteria: - Confirmed diagnosis of IBD (either CD or UC) with moderate disease - Age 18 years or older - All sexes eligible Exclusion criteria: - Pregnant, HIV-positive individuals - Use of immunosuppressive drugs (unless for IBD treatment) - Active cancer within the past 5 years - Ginger allergy Result: after 30 days - Decrease in inflammatory cells in the biopsy after treatment versus before treatment - Decrease in subjective symptoms	NCT04879810 [18]

## 4. Discussion

### 4.1. Mechanisms of Action: A Three-Step Cellular Uptake Framework

Understanding the mechanisms of action underlying extracellular vesicle–mediated drug delivery is essential for translating promising preclinical findings into clinically effective therapies. Unlike conventional nanocarriers, EVs exploit endogenous cellular communication pathways, enabling efficient cellular uptake, cargo protection, and intracellular trafficking. The therapeutic activity of drug-loaded EVs can be conceptualized as a sequence of interdependent biological events encompassing cellular uptake, endosomal escape, and intracellular cargo engagement.

The therapeutic performance of drug-loaded EVs depends on a sequence of interrelated processes: (i) cellular uptake into target cells, (ii) endosomal escape and intracytoplasmic release of cargo, and (iii) subsequent intracellular targeting and functional engagement of molecular pathways. Dissecting these mechanisms is essential for rational design and optimization of EV-based drug delivery systems.

#### 4.1.1. Step 1—Cellular Uptake

Drug-loaded EVs can enter target cells through multiple, partially overlapping endocytic pathways. The most frequently implicated mechanisms include clathrin-mediated endocytosis, macropinocytosis, and lipid raft/caveolin-dependent uptake. Clathrin-mediated endocytosis is often responsible for a substantial fraction of EV internalization (40–60% in several model systems), while macropinocytosis contributes significantly at higher EV concentrations (50–80% reduction with inhibitors). Lipid raft- and caveolin-dependent mechanisms are particularly important for smaller EVs (<100 nm) enriched in tetraspanins.

#### 4.1.2. Step 2—Endosomal Escape and Cargo Release

After internalization, EVs are typically trafficked to early endosomes and then along the endo-lysosomal pathway. For therapeutic cargo to exert intracellular effects, especially for nucleic acids and certain small molecules, efficient endosomal escape is required. Several escape mechanisms have been proposed, including back-fusion of internal EV membranes with limiting endosomal membranes, pH-dependent destabilization of membranes modulated by lipid composition and protein corona, and fusion events facilitated by fusogenic lipids or engineered peptides.

#### 4.1.3. Step 3—Cargo Targeting and Functional Engagement

The final step involves trafficking of released cargo to its intracellular site of action. EV surface proteins such as phosphatidylserine-binding moieties, integrins, and tetraspanins (CD9, CD63, CD81) can direct preferential uptake by specific cell types or tissues. Engineered decorations (e.g., aptamers, peptides, antibodies) can further refine targeting specificity. The interplay between intrinsic EV tropism and exogenous targeting ligands represents a key design variable.

### 4.2. Advantages and Limitations of EV-Based Drug Delivery

EV-based drug delivery offers several advantages over conventional approaches. First, EVs enhance biocompatibility and exhibit low immunogenicity due to their natural origin, making them less likely to trigger immune responses than synthetic nanoparticles. For instance, MSC-derived EVs show a low risk of immunogenicity and tumorigenicity compared to whole-cell therapy [5]. Second, EVs can encapsulate a wide range of therapeutic agents, including hydrophobic drugs such as curcumin [3,72]. Third, they enable targeted delivery to specific tissues or tumors [7].

A comparative overview of extracellular vesicle sources and their key characteristics across therapeutic applications is summarized in Table 1. For example, AS1411 aptamer-functionalized exosomes targeted nucleolin-positive colorectal cancer cells [6], while M2 macrophage-derived EVs accumulated at spinal cord injury sites [7].

However, EV-based delivery also faces notable limitations. Loading efficiency varies with both the drug and the loading method, requiring optimization of parameters; for instance, loading lonidamine into EVs resulted in markedly different encapsulation efficiencies—0.81 ± 0.22% and 4.16 ± 1.9%, respectively—despite using the same EV source and method [2]. Similarly, loading 17β-estradiol by passive versus active methods yielded different efficiencies [20]. In addition, the long-term safety profile and biodistribution of drug-loaded EVs are not fully established. In alopecia areata mouse models, baricitinib-loaded EVs reduced inflammation and promoted hair regrowth, but the authors noted the absence of long-term toxicological data and emphasized the need for further safety evaluation prior to clinical translation.

Additionally, large-scale manufacturing, batch-to-batch reproducibility, and quality control of EV formulations continue to pose challenges for regulatory standardization. Overall, EV-based systems represent a biologically inspired and highly adaptable platform for drug delivery, yet their clinical transition depends on addressing these safety, reproducibility, and scalability limitations [24].

Only a minority of the included studies reported exogenous loading of nucleic acids into EVs, typically in the context of co-delivery with chemotherapeutic agents (e.g., miRNA mimics or plasmid constructs combined with taxanes or other cytotoxics). These formulations aimed to synergistically modulate oncogenic signaling and chemosensitivity, but they remain at an early preclinical stage and were numerically outweighed by small-molecule-only EV formulations in the 2020–2025 literature set.

### 4.3. Disease-Specific Pathophysiological Targeting of Drug-Loaded EVs

The therapeutic relevance of drug-loaded EVs is highly dependent on disease-specific pathophysiological mechanisms. In oncology, EVs exploit enhanced permeability and retention effects, tumor-associated macrophage interactions, and receptor-mediated uptake to improve intratumoral drug accumulation while reducing systemic toxicity. In inflammatory and autoimmune disorders, EVs preferentially accumulate at inflamed sites due to increased vascular permeability and immune cell recruitment, enabling localized immunomodulation. In neurodegenerative diseases, the ability of EVs to cross the blood–brain barrier represents a decisive advantage, facilitating central nervous system drug delivery where conventional formulations fail.

### 4.4. Loading Method Preferences and Trade-Offs

Across the studies included, passive loading was the most frequently employed approach, likely due to its procedural simplicity and minimal disruption of vesicle integrity. Representative drug-loaded EV formulations, loading methods, and experimental conditions are summarized in Table 2.

This strategy was particularly effective for hydrophobic molecules, which readily partition into the lipid bilayer. For example, curcumin and berberine were successfully incorporated into EVs via passive incubation [7,8]. However, passive loading often results in low encapsulation efficiency, especially for hydrophilic or charged drugs. Li et al. reported a marked difference in loading outcomes between doxorubicin and lonidamine, despite using the same EV source and method, highlighting how drug properties influence efficiency [2].

In contrast, active loading methods—including electroporation, sonication, extrusion, and freeze–thaw cycles—were employed to overcome poor passive permeability. These techniques improved encapsulation of hydrophilic agents, such as doxorubicin and cisplatin [2,9], but also carried risks of membrane damage, aggregation, or altered surface protein composition, which may affect biodistribution and biological function. To balance these trade-offs, several studies employed mixed strategies that combined passive incubation with an active step, though outcomes remained highly variable and poorly standardized [20].

An overview of ongoing and completed clinical trials involving drug-loaded extracellular vesicles is provided in Table 3.

Despite encouraging preclinical outcomes, significant heterogeneity exists across studies regarding EV source selection, isolation protocols, loading efficiency, and biological characterization. Reported drug encapsulation efficiencies vary widely even for identical drugs and loading methods, reflecting both methodological inconsistencies and intrinsic EV variability. Furthermore, active loading techniques, while improving encapsulation of hydrophilic cargo, may compromise membrane integrity and surface protein composition, potentially altering biodistribution and biological function. These unresolved issues underscore the need for standardized protocols and comparative studies.

**Figure 2:**
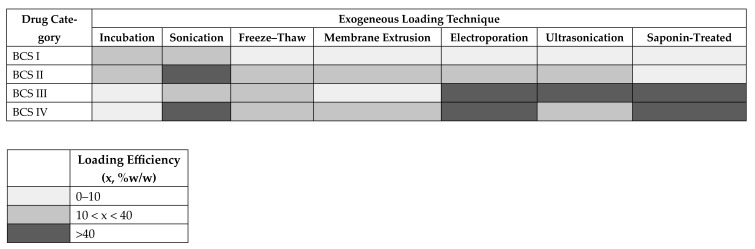
Matrix analysis loading efficiency across different exogenous loading techniques.

To further clarify these observations, we synthesized the data into a matrix analysis of loading efficiency across different exogenous loading techniques, stratified by Biopharmaceutics Classification System (BCS) drug categories (Table 4; Figure 2). The resulting heat matrix (Figure 2) visually captures these trade-offs between drug class and loading efficiency, providing a comparative framework to guide method selection. Passive approaches remain preferable for simple, lipophilic molecules where membrane insertion is sufficient, while active methods are indispensable for hydrophilic, charged, or bulky cargo. This analysis emphasizes that no single loading technique is universally optimal; rather, method choice should be tailored to the drug’s BCS profile while balancing encapsulation yield against preservation of vesicle structure.

To further clarify these observations, we synthesized the data into a matrix analysis of loading efficiency across different exogenous loading techniques, stratified by Biopharmaceutics Classification System (BCS) drug categories (Table 4; Figure 2).

The resulting heat matrix (Figure 2) visually captures these trade-offs between drug class and loading efficiency, providing a comparative framework to guide method selection.

Passive approaches remain preferable for simple, lipophilic molecules where membrane insertion is sufficient, while active methods are indispensable for hydrophilic, charged, or bulky cargo.

This analysis emphasizes that no single loading technique is universally optimal; rather, method choice should be tailored to the drug’s BCS profile while balancing encapsulation yield against preservation of vesicle structure.

In the future, the integration of microfluidic technologies, machine learning-based optimization, and standardized evaluation protocols may facilitate more reproducible and scalable EV-loading strategies.

**Table 4:** Drug class and loading efficiency.

BCS Class	Representative Drug	Preferred Loading Techniques	Observed Efficiency
Class I (High solubility, High permeability)	Estradiol [1], Aspirin [8], Delphinidin [36]	Incubation (passive), mild sonication	Estradiol: passive ~75 ng/100 µg vs. active ~60 ng/100 µg (incubation > sonication). Aspirin: low efficiency with sonication (0.587 µg/mL). Delphinidin: 9% with passive incubation. Overall: gentle methods sufficient, but efficiency is modest.
Class II (Low solubility, High permeability)	Curcumin [14,20,67,71], Berberine [11,12,37], Atorvastatin [6], Docetaxel [21,23,40,55,74], Paclitaxel [2]	Passive incubation, sonication, extrusion	Curcumin: 10% (incubation), 11% (sonication), 9% (freeze–thaw). Berberine: variable efficiency with passive + sonication (up to ~40%). Docetaxel: 12–18% with electroporation; ~77% with passive vortexing; very low (1.3%) with extrusion. Lipophilic drugs load well but are technique-dependent
Class III(High solubility, Low permeability)	Doxorubicin [4,5,7,24,25,26,41,46,48,56,57,62,68,73], Cisplatin [17], Insulin [42], Baricitinib [9]	Electroporation, ultrasonication, saponin, freeze–thaw	Doxorubicin: encapsulation efficiency ranged from <1% to ~89%, depending on method and excipients. Cisplatin: ~32% with incubation. Insulin: ~50% with electroporation. Baricitinib: 86 µg/mL with electroporation. Active methods clearly outperform passive incubation for hydrophilic/charged molecules.
Class IV(Low solubility, Low permeability; large/complex molecules)	Rapamycin [39,51], Amphotericin B [44], Bevacizumab (mAb) [50], Kaempferol [49], Luteolin [47]	Sonication, electroporation, saponin, freeze–thaw, extrusion	Bevacizumab: freeze–thaw (61 µg), co-incubation (65 µg), saponin (74 µg), sonication (61 µg). Luteolin: passive 3%, sonication 40%. Amphotericin B: large gains with active approaches (sonication, extrusion, electroporation). These complex/larger molecules require aggressive methods, with clear trade-offs in EV integrity.

### 4.5. Clinical Translation and Future Directions

Despite substantial progress in preclinical research, the clinical translation of drug-loaded extracellular vesicles remains limited, highlighting critical gaps in standardization, large-scale manufacturing, long-term safety evaluation, and regulatory harmonization. Recent systematic and narrative reviews catalog a substantially larger number of EV-related clinical trials, including unmodified EV therapeutics, diagnostic applications, and studies registered in multiple international repositories; our analysis should therefore be interpreted as a strictly defined subset concentrating on EVs with explicitly described exogenous drug loading. While EV-based therapy has emerged as a rapidly growing area of research, only 2 clinical trials were included in this review (NCT06930326 [19]; NCT04879810 [18]), indicating that most studies remain at the preclinical stage. This gap highlights the need for greater emphasis on translational studies that bridge laboratory-scale findings with clinical validation. A major hurdle in translating EVs to clinical use is the lack of standardized platforms for their production, measurement, and molecular characterization, including critical variability across laboratories. The absence of harmonized protocols for isolation, purification, and potency testing hampers the comparability and reproducibility of results across studies. Another key limitation involves targeting efficiency remaining suboptimal, and off-target delivery continues to limit therapeutic precision. Strategies such as surface ligand conjugation or charge modification have demonstrated potential to enhance circulation time and accumulation at target sites [79]. Further development of engineered EVs with tunable surface properties and controlled release kinetics may significantly improve clinical performance. Recent advances in bioengineered extracellular vesicles, including genetically modified producer cells and surface-functionalized vesicles, highlight promising strategies to enhance targeting specificity, improve loading efficiency, and facilitate clinical translation. A scalable manufacturing procedure and quality control for EVs is still a big challenge because of the biological complexity of EVs. Recent advances, including the use of bioreactor systems, have yielded up to a 38-fold increase in productivity compared with conventional flask cultures while preserving critical quality attributes [80]. However, unless such large-scale production methods become cost-effective, clinical use of EV-based therapies will likely remain confined to niche or experimental applications.

In the future, integrating automated bioprocessing, advanced analytics, and real-time quality control systems could enable the transition of EV-based drug delivery from bench to bedside. Collaborative standardization efforts among academia, industry, and regulatory bodies will be essential to ensure safe, reproducible, and scalable EV therapeutics.

## 5. Conclusions

EV-based drug delivery systems represent a promising next-generation therapeutic platform that combines the advantages of biological compatibility, versatile cargo loading, and targeted delivery.

The studies included in this review collectively demonstrate that EVs can effectively encapsulate and transport a wide range of therapeutic agents, enhancing stability, cellular uptake, and pharmacological efficacy compared with conventional nanocarriers. However, challenges related to standardized isolation methods, scalable manufacturing, and reproducible loading efficiency continue to hinder clinical translation. The integration of engineered EVs, automated bioprocessing, and advanced analytics is expected to improve reproducibility and facilitate regulatory approval in the future. A coordinated, multi-disciplinary research approach emphasizing standardization, manufacturing optimization, and clinical trial prioritization is required to advance EV-based therapeutics from bench to bedside. With sustained investment in research infrastructure, regulatory pathway clarity from FDA/EMA guidance documents, and collaborative academic-industry partnerships, EV-based therapeutics have the potential for substantial clinical impact across oncology, neurodegeneration, inflammatory diseases, and regenerative medicine applications.

Overall, EV-based formulations hold substantial potential to bridge the gap between experimental nanomedicine and clinical therapeutics, offering new opportunities for precision and regenerative medicine.

## Data Availability

No new data were created or analyzed in this study.

## Supplementary Materials

The following supporting information can be downloaded at: https://www.mdpi.com/article/10.3390/pharmaceutics18010045/s1, PRISMA 2020 checklist [81].

## Abbreviations

The following abbreviations are used in this manuscript:

EVs	Extracellular Vesicles
