# Supplementary material for: Drug-Loaded Extracellular Vesicle-Based Drug Delivery: Advances, Loading Strategies, Therapeutic Applications, and Clinical Challenges
Source: Pharmaceutics. 2025 Dec 29;18(1):45. doi: 10.3390/pharmaceutics18010045 (PMC12845221; doi:10.3390/pharmaceutics18010045)
Supplement: Supplementary file 1 [file pharmaceutics-18-00045-s001.zip › pharmaceutics-4014573-supplementary.pdf]

PRISMA 2020 Checklist

Section and Topic	Item #	Checklist item	Location where item is reported
**TITLE**			
Title	1	Identify the report as a systematic review.	Title page; Abstract (Background-Objectives)
**ABSTRACT**			
Abstract	2	See the PRISMA 2020 for Abstracts checklist.	Abstract section; includes Background, Objectives, Methods, Results, Conclusions keywords
**INTRODUCTION**			
Rationale	3	Describe the rationale for the review in the context of existing knowledge.	Section 1. Introduction (entire section); explains gap in clinical translation of drug-loaded EVs and need for systematic synthesis (2020-2025)
Objectives	4	Provide an explicit statement of the objective(s) or question(s) the review addresses.	Section 1. Introduction (final paragraph); review aims to summarize EV sources, loading strategies, therapeutic applications, and translational challenges
**METHODS**			
Eligibility criteria	5	Specify the inclusion and exclusion criteria for the review and how studies were grouped for the syntheses.	Section 2.1 Eligibility Criteria; includes inclusion of original research articles on drug-loaded EVs from human/animal/plant sources, exclusion of reviews/editorials/abstracts; study period 2020-2025; English language
Information sources	6	Specify all databases, registers, websites, organisations, reference lists and other sources searched or consulted to identify studies. Specify the date when each source was last searched or consulted.	Section 2.2 Search Strategy; databases: Embase, PubMed, Reaxys, Scopus, and ClinicalTrials.gov; search period: 2020-2025
Search strategy	7	Present the full search strategies for all databases, registers and websites, including any filters and limits used.	Section 2.2 Search Strategy; keywords: "Extracellular vesicles AND drug delivery OR drug-loaded extracellular vesicles"; English language

PRISMA

PRISMA 2020 Checklist

Section and Topic	Item #	Checklist item	Location where item is reported
			limit; date range 2020-2025
Selection process	8	Specify the methods used to decide whether a study met the inclusion criteria of the review, including how many reviewers screened each record and each report retrieved, whether they worked independently, and if applicable, details of automation tools used in the process.	Section 2.3 Screening process and data extraction; records imported to Endnote, duplicates removed, title/abstract screening followed by full-text review of eligible studies
Data collection process	9	Specify the methods used to collect data from reports, including how many reviewers collected data from each report, whether they worked independently, any processes for obtaining or confirming data from study investigators, and if applicable, details of automation tools used in the process.	Section 2.3 Screening process and data extraction; data organized into three structured tables (Table 1, 2, 3); extracted data includes EV source, drug type, particle size, loading method, administration route, therapeutic indication, loading efficiency, and clinical trial details
Data items	10a	List and define all outcomes for which data were sought. Specify whether all results that were compatible with each outcome domain in each study were sought (e.g. for all measures, time points, analyses), and if not, the methods used to decide which results to collect.	Section 2.3 and Results section; variables include: EV source type, drug type, particle size (nm), targeted release, administration route, drug release characteristics, indication, loading method, loading efficiency (%), loading conditions, outcomes, and clinical trial status
	10b	List and define all other variables for which data were sought (e.g. participant and intervention characteristics, funding sources). Describe any assumptions made about any missing or unclear information.	The missing information indicated as no information was available.
Study risk of bias assessment	11	Specify the methods used to assess risk of bias in the included studies, including details of the tool(s) used, how many reviewers assessed each study and whether they worked independently, and if applicable, details of automation tools used in the process.	Section 4.1 Discussion - Advantages and Limitations; notes variability in drug loading efficiency and safety profile gaps; acknowledges limitations in standardization across studies
Effect measures	12	Specify for each outcome the effect measure(s) (e.g. risk ratio, mean difference) used in the synthesis or presentation of results.	Section 3.2 Results - Tables 1, 2, 4; reports

PRISMA 2020 Checklist

Section and Topic	Item #	Checklist item	Location where item is reported
			loading efficiency percentages, encapsulation efficiency ranges, particle size ranges, and therapeutic outcomes
Synthesis methods	13a	Describe the processes used to decide which studies were eligible for each synthesis (e.g. tabulating the study intervention characteristics and comparing against the planned groups for each synthesis (item #5)).	Section 4.2 Loading Method Preferences and Trade-offs; describes synthesis into BCS classification matrix analysis (Table 4, Figure 2); compares passive vs. active loading methods across drug classes
	13b	Describe any methods required to prepare the data for presentation or synthesis, such as handling of missing summary statistics, or data conversions.	
	13c	Describe any methods used to tabulate or visually display results of individual studies and syntheses.	
	13d	Describe any methods used to synthesize results and provide a rationale for the choice(s). If meta-analysis was performed, describe the model(s), method(s) to identify the presence and extent of statistical heterogeneity, and software package(s) used.	
	13e	Describe any methods used to explore possible causes of heterogeneity among study results (e.g. subgroup analysis, meta-regression).	
	13f	Describe any sensitivity analyses conducted to assess robustness of the synthesized results.	
Reporting bias assessment	14	Describe any methods used to assess risk of bias due to missing results in a synthesis (arising from reporting biases).	Not reported
Certainty assessment	15	Describe any methods used to assess certainty (or confidence) in the body of evidence for an outcome.	Section 4.1 Discussion; discusses limitations including standardization gaps, off-target delivery challenges, scalability issues, and lack of long-term safety data
**RESULTS**			
Study selection	16a	Describe the results of the search and selection process, from the number of records identified in the search to the number of studies included in the review, ideally using a flow diagram.	Section 3.1 Study Selection and Figure 1; total 5,316 articles identified (Embase 2,968; PubMed 12; Reaxys 889; Scopus 1,447); 64 articles included after screening; 2 clinical trials included; PRISMA 2020 flow diagram presented
	16b	Cite studies that might appear to meet the inclusion criteria, but which were excluded, and explain why they were excluded.	A clinical trial investigating EVs as a biomarker in Alzheimer's disease: Excluded because it did not involve drug loading.<br>An original research article reporting EVs for cancer diagnosis and prognosis, without drug delivery

Section and Topic	Item #	Checklist item	Location where item is reported
			experiments: Excluded because of lack of active drug loading process.<br>A protocol for a planned study involving EV-loaded gene therapy with no published results by 2025: Excluded due to unavailable outcomes and incomplete study status
Study characteristics	17	Cite each included study and present its characteristics.	Section 3.2 Results of the study; Table 1 summarizes 65 drug-loaded EV studies with sources, drugs, particle sizes, routes, and indications; Table 2 details loading methods and efficiencies; Table 3 summarizes 2 clinical trials (NCT06930326, NCT04879810)
Risk of bias in studies	18	Present assessments of risk of bias for each included study.	Not reported
Results of individual studies	19	For all outcomes, present, for each study: (a) summary statistics for each group (where appropriate) and (b) an effect estimate and its precision (e.g. confidence/credible interval), ideally using structured tables or plots.	Section 3.2 Results - Tables 1-4 and Figure 2; presents individual study data on EV sources, drugs, loading methods, efficiencies; synthesis shows passive loading predominance (especially hydrophobic drugs) and active method superiority for hydrophilic cargo
Results of syntheses	20a	For each synthesis, briefly summarise the characteristics and risk of bias among contributing studies.	Section 4.2 and Table 4, Figure 2; presents BCS classification analysis of loading efficiency across exogenous loading techniques (incubation, sonication, freeze-thaw, extrusion, electroporation, ultrasonication, saponin); shows efficiency ranges by drug class I-IV
	20b	Present results of all statistical syntheses conducted. If meta-analysis was done, present for each the summary estimate and its precision (e.g. confidence/credible interval) and measures of statistical heterogeneity. If comparing groups, describe the direction of the effect.	
	20c	Present results of all investigations of possible causes of heterogeneity among study results.	
	20d	Present results of all sensitivity analyses conducted to assess the robustness of the synthesized results.	

PRISMA 2020 Checklist

Section and Topic	Item #	Checklist item	Location where item is reported
Reporting biases	21	Present assessments of risk of bias due to missing results (arising from reporting biases) for each synthesis assessed.	Section 4.1 Discussion; acknowledges that only 2 clinical trials found vs. 65 preclinical studies, indicating potential reporting bias and gap in translational research
Certainty of evidence	22	Present assessments of certainty (or confidence) in the body of evidence for each outcome assessed.	Section 4.1 Discussion - Advantages and Limitations; discusses evidence limitations: variability in loading efficiency dependent on drug and method; insufficient long-term safety data; lack of standardization; scalability challenges
**DISCUSSION**			
Discussion	23a	Provide a general interpretation of the results in the context of other evidence.	Section 4 Discussion (entire); compares EV advantages (biocompatibility, low immunogenicity) with limitations (loading efficiency variability, safety gaps); contextualizes within broader nanomedicine field
	23b	Discuss any limitations of the evidence included in the review.	
	23c	Discuss any limitations of the review processes used.	
	23d	Discuss implications of the results for practice, policy, and future research.	
**OTHER INFORMATION**			
Registration and protocol	24a	Provide registration information for the review, including register name and registration number, or state that the review was not registered.	Methods: PRISMA followed; **not registered** This systematic review was not registered in a public protocol registry (such as PROSPERO), as the protocol evolved during the data synthesis process to accommodate recent developments and methodological heterogeneity in the fast-moving field of drug-loaded extracellular vesicles. All eligibility criteria, search strategies, and screening steps are fully reported

PRISMA 2020 Checklist

Section and Topic	Item #	Checklist item	Location where item is reported
			within the manuscript in accordance with PRISMA 2020 guidelines.
	24b	Indicate where the review protocol can be accessed, or state that a protocol was not prepared.	Not available
	24c	Describe and explain any amendments to information provided at registration or in the protocol.	Not applicable
Support	25	Describe sources of financial or non-financial support for the review, and the role of the funders or sponsors in the review.	No financial or non-financial support were received.
Competing interests	26	Declare any competing interests of review authors.	No competing interests exist.
Availability of data, code and other materials	27	Report which of the following are publicly available and where they can be found: template data collection forms; data extracted from included studies; data used for all analyses; analytic code; any other materials used in the review.	The complete data extracted from included studies are presented as Tables 1, 2, and 3 within the manuscript itself. These tables summarize:<br>Study characteristics (EV source, drug type, particle size, administration route, indication, reference)<br>Loading strategies and efficiencies<br>Clinical trials on drug-loaded EVs<br>This data is publicly available as part of the manuscript and not in a separate file or external repository

From: Page MJ, McKenzie JE, Bossuyt PM, Boutron I, Hoffmann TC, Mulrow CD, et al. The PRISMA 2020 statement: an updated guideline for reporting systematic reviews. BMJ 2021;372:n71. doi: 10.1136/bmj.n71.
This work is licensed under CC BY 4.0. To view a copy of this license, visit https://creativecommons.org/licenses/by/4.0/
